# Health literacy time trends in Germany: Eva-Maria Berens

**DOI:** 10.1093/eurpub/ckac129.722

**Published:** 2022-10-25

**Authors:** D Schaeffer, J Klinger, E-M Berens, K Hurrelmann

**Affiliations:** 1 Institute for Sociology and Social Psychology, University of Cologne, Köln, Germany; 2 Interdisciplinary Centre for Health Literacy Research, University Bielefeld, Bielefeld, Germany; 3 Hertie School Berlin, Berlin, Germany

## Abstract

**Background:**

Health literacy (HL) is becoming increasingly important in the European public health sector. Since the Health information landscape is changing constantly there is a need for monitoring HL regularly in order to observe the influence of these changes, to enable trend statements and to obtain precise information on the societal and individual factors. For the first time, this has been done for the population in Germany.

**Methods:**

Cross-sectional data collected in the adult population in Germany in 2014 (n = 1.940) and 2020 (n = 504) was used. The instrument HLS-EU-Q47 was used to measure HL in the domains of health care, disease prevention and health promotion. Changes between time points were analysed on the population level as well as in several population groups.

**Results:**

The HL of the German population has become lower within the observed six years. This is evident in all three domains but is most pronounced in health promotion literacy. This trend is particularly visible among people with low social status and financial deprivation. Single health information tasks that are precepted as more difficult involve the evaluation of information and handling information from the media.

**Conclusions:**

Over time, dealing with health and disease-related information has become more difficult. As this development is mainly driven by socioeconomically disadvantaged population groups, it has apparently increased social inequality in the health sector. Due to more complex and demanding health information, which lately result in an ‘infodemic’, it seems that these groups in particular are overburdened and cannot raise the resources and skills to adequately engage with health information.

**Key messages:**

• HL interventions should focus on socioeconomically disadvantaged population groups to counteract the increasing health inequality.

• Monitoring HL should be included in public health surveillance to determine needs and challenges for health policies as well as achievements of interventions.

